# Ovarian Cancer and Parkinson’s Disease: A Bidirectional Mendelian Randomization Study

**DOI:** 10.3390/jcm12082961

**Published:** 2023-04-19

**Authors:** Jian-Zeng Guo, Qian Xiao, Lang Wu, Fa Chen, Jia-Li Yin, Xue Qin, Ting-Ting Gong, Qi-Jun Wu

**Affiliations:** 1 Department of Clinical Epidemiology, Shengjing Hospital of China Medical University, Shenyang 110004, China; 2 Department of Obstetrics and Gynecology, Shengjing Hospital of China Medical University, Shenyang 110004, China; 3 Cancer Epidemiology Division, Population Sciences in the Pacific Program, University of Hawaii Cancer Center, University of Hawaii at Manoa, Honolulu, HI 96813, USA; 4 Fujian Provincial Key Laboratory of Environment Factors and Cancer, Department of Epidemiology and Health Statistics, School of Public Health, Fujian Medical University, Fuzhou 350122, China; 5 Clinical Research Center, Shengjing Hospital of China Medical University, Shenyang 110004, China; 6 Key Laboratory of Precision Medical Research on Major Chronic Disease, Shengjing Hospital of China Medical University, Shenyang 110004, China

**Keywords:** genetic association, histotype, mendelian randomization, ovarian cancer, Parkinson’s disease

## Abstract

(1) Background: Ovarian cancer (OC) and Parkinson’s disease (PD) represent a huge public health burden. The relationship of these two diseases is suggested in the literature while not fully understood. To better understand this relationship, we conducted a bidirectional Mendelian ran-domization analysis using genetic markers as a proxy. (2) Methods: Utilizing single nucleotide polymorphisms associated with PD risk, we assessed the association between genetically predicted PD and OC risk, overall and by histotypes, using summary statistics from previously conducted genome-wide association studies of OC within the Ovarian Cancer Association Consortium. Similarly, we assessed the association between genetically predicted OC and PD risk. The inverse variance weighted method was used as the main method to estimate odds ratios (OR) and 95% confidence intervals (CI) for the associations of interest. (3) Results: There was no significant association between genetically predicted PD and OC risk: OR = 0.95 (95% CI: 0.88–1.03), or between genetically predicted OC and PD risk: OR = 0.80 (95% CI: 0.61–1.06). On the other hand, when examined by histotypes, a suggestive inverse association was observed between genetically predicted high grade serous OC and PD risk: OR = 0.91 (95% CI: 0.84–0.99). (4) Conclusions: Overall, our study did not observe a strong genetic association between PD and OC, but the observed potential association between high grade serous OC and reduced PD risk warrants further investigation.

## 1. Introduction

Cancer and neurodegenerative diseases represent chronic physiological ailments. As cancer progresses, proliferative signaling persists, growth suppressors are evaded, cell death is resisted, replicative immortality is acquired, angiogenesis is activated, and further, invasion and metastasis are activated [1,2,3,4]. A neurodegenerative disorder involves the loss of neurons, impairment of synaptic plasticity, proteinopathies, and other abnormalities [5], which include misfolded amyloid-β and tau in Alzheimer’s disease, α-synuclein in Parkinson’s disease (PD) [6,7,8], as well as progressive muscle atrophy or muscle wasting. As a result of neurodegeneration, people suffer memory loss, cognitive problems, and movement disorders [5]. Initially, the two fields do not seem to have much in common. Nevertheless, as we further understand these two types of diseases, we become more aware of their unexpected overlap. With the in-depth study of neurodegenerative diseases and cancer, a number of molecular mechanisms appear to operate inversely in cancer and neurodegenerative diseases, researchers are gradually discovering, leading either to resistance to cell death, or to increased cell death, respectively [9]. As well as sharing genetic and cellular alterations, neurodegeneration and cancer are also affected by common molecular pathways, such as autophagy and cell cycle regulation [10,11,12]. Recent meta-analyses of transcriptomic data have also shown significant overlap between genes that are up-regulated in neurodegenerative diseases and genes that are down-regulated in cancer, and vice versa [13]. Therefore, it is necessary to understand the association between specific cancers and specific neurodegenerative diseases. In this study, we wanted to explore the potential association between ovarian cancer (OC) and PD.

OC is the most common gynecological tumor with the highest mortality rate, the seventh most common cancer, and the eighth leading cause of cancer death in women, mostly occurring in postmenopausal women over the age of 50 [14]. The symptoms of the disease are usually not obvious, it is diagnosed at an advanced stage, and 75% of cases have spread by the time of clinical diagnosis [15]. The survival of OC patients is related to the stage at diagnosis. For example, in the United States, a small proportion of patients with stage I ovarian cancer have a 5-year survival rate of more than 90%. The 5-year survival rate is 75% to 80% for patients with local tumors and only 25% for those with distant metastases. Despite the favorable prognosis of early OC, the overall 5-year survival rate is only 48.6%, so there is an urgent need to develop effective prevention strategies to reduce the public health burden of OC [16,17]. In recent years, factors influencing OC risk have included hereditary risk factors (including absence of pregnancy, early age of menarche, and late age at menopause), smoking, benign gynecological conditions (including endometriosis, polycystic ovary syndrome, and pelvic inflammatory disease), and possibly talcum powder use [18,19]. In contrast, few studies have explored the association between ovarian cancer and neurodegenerative diseases, particularly Parkinson’s disease.

PD is the most common type of degenerative motor disease of the central nervous system and the most common disease with symptoms of movement disorders [20]. In terms of number of people affected, the disease is a common condition, affecting an estimated 6.1 million people globally in 2016 [21]. For reasons that are not yet fully understood, the incidence and prevalence of this disease have increased rapidly over the past two decades [22,23]. PD has a profound impact on both patients and caregivers. The disease duration characteristic of the degenerative disease can last for decades, and most caregivers experience excessive stress [24]. For society, PD poses an increasing socioeconomic burden [25]. Therefore, to understand the pathogenesis of PD and find effective prevention and treatment methods is not only a major problem to be solved in the medical field, but also an extremely serious sociological problem [26]. When discussing the etiology of PD, the three factors of genetics, environment, and their interaction are often mentioned together [27]. It is worth mentioning that some molecules that have received extensive attention in cancer-related studies, such as the Bcl-2 family, caspase family, tumor necrosis factor-α (TNF-α), Parkin and phosphate and tension homology deleted on chromosome ten (PTEN) induced putative kinase 1 (PINK1), are closely related to the development and progression of PD. However, dedicated reports on the relationship between OC and PD have remained sparse. We consider these areas of convergence and examine how insight and understanding from one disease to the other may help us prevent versus treat.

Recently, the results of two meta-analyses from observational studies showed no association between PD and OC risk [28,29]. However, the third meta-analysis noted that patients with PD had a lower prevalence of OC [30]. Inconsistent findings from meta-analyses often stem from differences in the included cohorts and confounding factors in the studies. On this basis, conflicting results may also be reported when identifying and adjusting for various potential confounding factors. Further, when studying the association between two diseases, it is difficult to model the disease through interventions due to technical limitations and ethical constraints. As a result, the vast majority of previous studies on associations between diseases were observational. The results (associations) obtained from observational studies are often accompanied by a lack of causal effects. The use of genetic variants as instrumental variables (IVs) has been suggested as a way to overcome the limitations of observational design, usually single-nucleotide polymorphisms (SNPs), to simulate how modifiable environmental exposures affect disease susceptibility, known as Mendelian randomization (MR) [31]. In the MR study, researchers first identified and extracted SNP information associated with exposure at the genome-wide significance level (*p* = 5 × 10^−8^) and then used these SNPs as IV to assess the relationship between exposure and outcome to obtain an odds ratio (OR) and mean differences [32]. To further understand the bidirectional association between PD and OC and to clarify the causal relationship, we chose a MR approach.

## 2. Materials and Methods

### 2.1. Standard Protocol Approvals, Registrations, and Patient Consents

This study used summary data published by multiple GWAS; patient consents were obtained by corresponding studies. This study is reported following the Strengthening the Reporting of Observational Studies in Epidemiology reporting guideline.

### 2.2. Selection of Genetic Instrumental Variables

In total, PD, OC, and six clinically common phenotypes of OC (high grade serous OC [HGSOC], low grade serous OC [LGSOC], endometrioid OC [EndoOC], clear cell OC [CCOC], mucinous OC [MOC], and low malignant potential OC [LMPOC]) were considered in this study. The following criteria were taken when obtaining IVs: r^2^ < 0.001; kb = 10,000; *p* < 5 × 10^−8^.

### 2.3. Ovarian Cancer Data

Results from a genome-wide association study (GWAS) as part of the Ovarian Cancer Association Consortium (OCAC) were used to determine the association between 25,509 OC cases and 40,941 controls of European descent [33]. This dataset comprises of 63 genotyping project/case–control sets. Genotype data were obtained by direct genotyping using an Illumina Custom Infinium array (OncoArray) consisting of approximately 530k SNPs, with imputation performed with reference to the 1k Genomes Project Phase 3 dataset [34]. A total of 25,509 OC cases and 40,941 controls were included. The cases involved the following invasive epithelial OC histotypes: HGSOC (*n* = 13,037), LGSOC (*n* = 1012), MOC (*n* = 1417), EndoOC (*n* = 2810), and CCOC (*n* = 1366). Invasive histotypes classified as “other” by OCAC (*n* = 2764 cases) were included in analyses of overall invasive epithelial OC but not assessed separately. Analyses were also performed for low malignant potential tumors (*n* = 3103), which included 1954 serous and 1149 mucinous tumors [33].

### 2.4. Parkinson’s Disease Population

GWAS summary results were obtained from 37,688 cases, 18,618 UKB proxy-cases and 1,417,791 controls at 7,784,415 SNPs of European descent in the International Parkinson’s Disease Genomics Consortium [35]. Using a single-stage design and a meta-analysis of all available aggregated GWAS data, 90 independent genome-wide significant signals were identified from 78 loci, including 38 independent risk signals from 37 novel loci. It has been found that these variants explain between 26% and 36% of the heritable risk of PD. Single cell expression data indicate that PD loci are heavily brain-enriched, consistent with specific neuronal cell types being implicated from tissue expression enrichment analyses.

### 2.5. Mendelian Randomization

**Figure 1:**
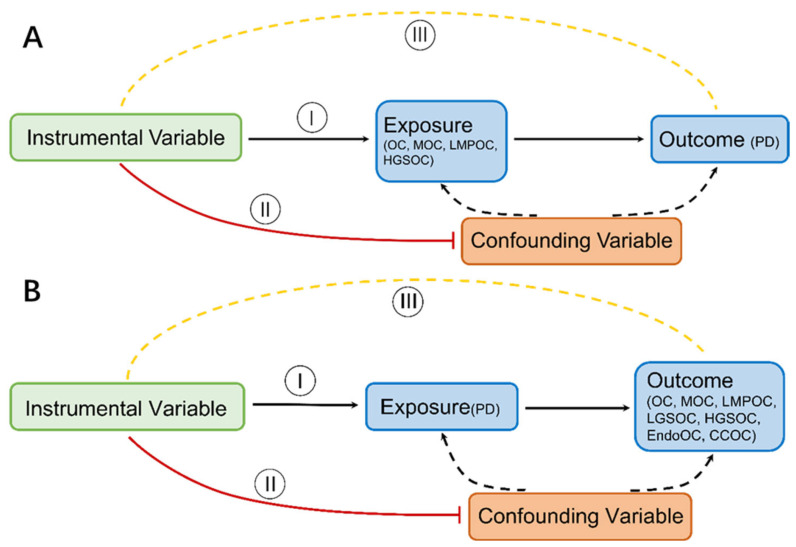
MR concepts and underlying IV assumptions are shown in a directed acyclic graph (I–III). (**A**) OC and its subtypes on the risk of PD (**B**) PD on the risk of OC and its subtypes.

For the main MR analysis, the inverse-variance weighted (IVW) method was used. This method assumes the absence of invalid genetic variables (e.g., pleiotropy) when summarizing genetic information to estimate the association between exposure and outcome [36]. A fixed-effect IVW meta-analysis of the Wald ratios (gene-outcome [log OR] divided by gene exposure relationships) estimated for each instrumental variable was used to derive the mean effect estimate from each outcome database individually [37]. Findings are presented as the odds ratio (OR) for the risk of the event given a change in the exposure. This OR is an estimate of the causal influence of the exposure on outcome when the MR assumptions are true. The following justifies the MR and necessary IV assumptions: I. The IVs (SNPs being employed) should have a good correlation with the exposure(s) under consideration. II. Confounding variables should not in any manner be connected to IVs. III. Only the relevant exposure(s) should be used to relate IVs to outcomes (Figure 1). We used weighted median estimation, MR-Egger regression analysis, and the MR pleiotropy residual sum and outlier (MR-PRESSO) method to look for any directional pleiotropy-related violations of the main MR hypothesis [38]. In MR-Egger, the intercept estimates the mean pleiotropy of genetic variants; a non-zero value indicates a biased IVW estimate [39]. The horizontal pleiotropy was additionally detected and adjusted using the MR-PRESSO approach by removing outliers [40]. If at least half of the instrumental variables are valid, the weighted median estimation analysis yields reliable and accurate results [41]. The Q statistic was also used to check for variability among calculated Wald ratios from various genetic variations [42]. The R packages “TwoSampleMR” and “MRPRESSO” were used to conduct the analysis (R version 4.0.5).

## 3. Results

Summary information of instruments identified for PD, OC and its common subtypes are presented in Supplemental Tables S1–S5. A total of 20 SNPs associated with PD risk were identified from 33,674 cases in a GWAS. (Nalls et al., 2019) Twenty SNPs associated with OC were identified from 25,509 cases in another above mentioned GWAS. (Phelan et al., 2017b) For HGSOC, 11 SNPs were identified from 13,037 cases. For LMPOC, four SNPs were identified from 3103 cases. For MOC, three variants were identified from 2566 cases. For LGSOC, EndoOC, and CCOC, there was no significantly associated variant available for MR analysis.

### 3.1. Effect of OC on PD

**Figure 2:**
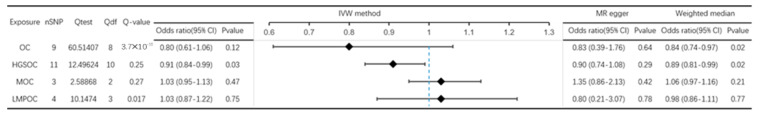
MR analysis results from OC to PD risk. IVW method, MR egger, and weighted median method estimate for the association of OC (including its common subtypes) with risk of PD.

For each assessed exposure, the number of SNPs included in the corresponding genetic instrument, along with Q statistics for the instrument, are provided in Figure 2. When using OC and its common subtypes as exposures, there was suggestive evidence for an association between HGSOC and risk of PD (OR = 0.91, 95% Confidence Interval (CI): 0.84–0.99 in IVW model, and OR = 0.89, 95% CI: 0.81–0.99 in weighted median estimator (Figure 2). However, the MR-Egger results (OR = 0.90, 95% CI: 0.74–1.08) indicated that horizontal pleiotropy might exist in this trial. However, no effect of horizontal pleiotropy was found in MRPRESSO (*p*-value = 0.216). Overall, in the primary analyses using IVW, except for HGSOC, genetically determined OC and its common subtypes were not associated with the risk of PD (for OC, OR = 0.80, 95% CI: 0.61–1.06; for MOC, OR = 1.03, 95% CI: 0.95–1.13; for LMPOC, OR = 1.03, 95% CI: 0.87–1.22) (Figure 2). No meaningful results were found in subsequent MR egger and median weighted analyses. The effects of single SNPs on OC and its common subtypes are summarized in Supplementary Tables S1–S4. The results of the leave-one-out analysis and the linear regression estimates of the five MR methods are detailed in the Supplementary Figures S1–S4.

### 3.2. Effect of PD on OC

**Figure 3:**
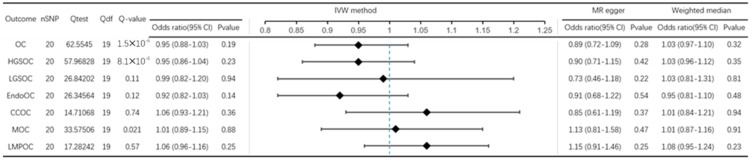
MR analysis results from PD to OC risk. IVW method, MR egger and weighted median method estimate for the association of PD with risk of OC (including its common subtypes).

When using PD as exposure, the overall association between genetically predicted PD and OC risk was 0.95 (95% CI: 0.88–1.03) (Figure 3). When results were examined by OC subtypes, genetically determined PD were not associated with the risk of any common subtypes of OC (for HGSOC, OR = 0.95, 95% CI: 0.86–1.04; for LGSOC, OR = 0.99, 95% CI: 0.82–1.20; for EndoOC, OR = 0.92, 95% CI: 0.82–1.03; for CCOC, OR = 1.06, 95% CI: 0.93–1.21; for MOC, OR = 1.01, 95% CI: 0.89–1.15; for LMPOC, OR = 1.06, 95% CI: 0.96–1.16) (Figure 3). Similarly, no meaningful results were found in subsequent MR egger and weighted median analyses. The effects of single SNPs on PD are summarized in Supplementary Tables S5–S11. The results of the leave-one-out analysis and the linear regression estimates of the five MR methods are detailed in the Supplementary Figures S5–S11.

## 4. Discussion

This MR analysis showed little unambiguous evidence to support roles of genetic liability to OC and PD in each other’s etiology. There was encouraging evidence for associations between genetic vulnerabilities to HGSOC and decreased PD risk in analyses looking at invasive epithelial OC histotypes and low malignant potential tumors. The MR approach, which is known to lessen some biases present in observational research and has the potential to produce evidence in support of causal correlations, is being used for the first time in this study to investigate the relationship between OC and its prevalent subtypes and PD status.

The results on OC in this study are in line with some earlier observational studies. For instance, a meta-analysis found no significant link between PD and the risk of OC, but a substantial inverse association between PD and the risk of all malignancies, digestive system cancers, lung cancer, and urological cancers [28]. A study of the Taiwanese population, on the other hand, showed an increased risk of cancer in PD patients. Among the 19 cancers, there was no association between PD and OC risk [29]. However, another meta-analysis reported different results, which suggested an inverse relationship between PD and OC [30]. The different results of these previous studies, in addition to differences in the included cohorts, may be due to uncontrolled confounding bias. In the end, no observational study can entirely eliminate residual confounding, and it is challenging to identify the factors that should be regarded as confounders of the link between OC and PD. On the other hand, commonalities in PD and OC regarding molecular mechanisms such as protein misfolding and degradation, cell cycle regulation and apoptosis, mitochondria and oxidative stress, and Phosphoinositide 3-Kinase (PI3K)- Protein Kinase B (AKT)- mammalian target of rapamycin (mTOR) signaling pathway have been widely noted [43]. With the completion of large GWAS, some genes that are altered in PD have been identified in OC, such as *SNCA* (encoding PARK1 and PARK4, expressed in OC but not in normal ovarian tissue) [44], Ubiquitin carboxyl-terminal hydrolase isozyme L1 (UCHL1)(encoding PARK5, associated with p53 ubiquitination, silenced in OC) [45], *PINK* (encoding PARK6, heterozygous mutation in OC), and *ATP13A2* (encoding PARK9, missense mutation in OC) [43].

Based on these aspects, we chose the MR method to explore the associations between PD and OC. Due to the nature of MR, when evaluating the correlation between the genetic tool for OC and PD risk, confounding bias is less likely to be present. Population stratification is a significant potential confounder that could affect the relationship between genetic variations and outcomes. In our main analysis, we only included people with European ancestry as a means of population stratification adjustment. In addition, our findings are based on the use of previous GWAS with various subpopulations, which strengthens our findings.

### 4.1. Study Strengths

There are two main strengths in the present study. First, the MR analysis is mainly based on reuse of data of GWAS, allowing re-analyzing existing GWAS data to explore potential causal associations between PD and OC. Due to the random assignment of alleles during meiosis, MR studies are relatively immune to common behavioral, physiological, and socioeconomic confounders, if all assumptions are met [46]. In many cases, genetic variation is precisely measured and reported, and therefore could be free of bias and error, which is particularly useful for assessing risk factors for long-term effects [47]. Thus, MR is particularly informative for assessing potential causal effects [48]. Second, this study is the first to investigate the potential causal associations between PD and OC (including its common subtypes) using MR methods. Such analyses leverage the large GWAS data involving large numbers of disease cases and controls, which is an effective extension of previous research.

### 4.2. Study Limitations

There are several limitations in the present study. First, for assessing the association of HGSOC with PD risk, the trends we observed in the IVW method and the MR Egger method are not exactly the same. The results of the IVW method showed that HGSOC was associated with a reduced risk of PD, but this association was not statistically significant in the MR Egger results. This indicates the possible presence of horizontal pleiotropy among the IVs used. Furthermore, we did not find the effect of horizontal pleiotropy on the results obtained by the IVW method in the subsequent MRPRESSO test. Second, the GWAS for PD were not stratified by sex, while the summary statistics for OC were only in females. This may be due to several observations suggesting that PD may not be a single entity. Clinical syndromes with phenotypically similar symptoms can arise from a variety of causes [49]. Even when a specific cause is found, the symptoms and course of PD frequently display a highly variable pattern [50]. Each PD patient has different priorities, needs, and wishes. As a result, every person has a relatively individual form of PD, making stratification in PD-related GWAS studies difficult to complete [27]. Further studies are therefore needed to confirm the results by using PD GWAS summary statistics focusing on women. Unfortunately, additional data sets or cohorts that can be scaled up are not yet available, and perhaps more convincing significant associations may emerge as additional data become available. Third, in this study, only three and four instrumental SNPs were used as instrumental variables when analyzing the associations of MOC and LMPOC on PD risk, respectively. Although these SNPs explain some of the variation in MOC and LMPOC, MR analysis would yield more robust results if additional independent instrumental variables could be used which can explain larger proportion of variance of the exposures. Fourth, the present study only analyzed the associations from a genetic perspective using SNPs as instrumental variables, and more in-depth exploration remains to be done.

## 5. Conclusions

In summary, using a comprehensive MR study design, we found little evidence sup-porting causal associations between OC and PD overall. Our results, suggesting that genetically predicted HGSOC may be associated with reduced risk of PD, warrant further investigation.

## Data Availability

The datasets analyzed for this study are publicly available and accessed as described below. No new data were generated. (See Supplementary Table S12 for GWAS IDs, etc.) GWAS data are accessed through the OpenGWAS project provided by the MRC Integrative Epidemiology Unit at the University of Bristol at: https://gwas.mrcieu.ac.uk (5 May 2020).

## Supplementary Materials

The following supporting information can be downloaded at: https://www.mdpi.com/article/10.3390/jcm12082961/s1, Supplementary Figure S1: (A) Leave-one-out sensitivity analysis for Ovarian cancer on the Parkinson’s disease. (B) Scatter plot of the association between Ovarian cancer and Parkinson’s disease; Supplementary Figure S2: (A) Leave-one-out sensitivity analysis for Mucinous ovarian cancer on the Parkinson’s disease. (B) Scatter plot of the association between Mucinous ovarian cancer and Parkinson’s disease.; Supplementary Figure S3: (A) Leave-one-out sensitivity analysis for Low malignant potential ovarian cancer on the Parkinson’s disease. (B) Scatter plot of the association between Low malignant potential ovarian cancer and Parkinson’s disease; Supplementary Figure S4: (A) Leave-one-out sensitivity analysis for High grade serous ovarian cancer on the Parkinson’s disease. (B) Scatter plot of the association between High grade serous ovarian cancer and Parkinson’s disease; Supplementary Figure S5: (A) Leave-one-out sensitivity analysis for Parkinson’s disease on the Ovarian cancer. (B) Scatter plot of the association between Parkinson’s disease and Ovarian cancer; Supplementary Figure S6: (A) Leave-one-out sensitivity analysis for Parkinson’s disease on the Mucinous ovarian cancer. (B) Scatter plot of the association between Parkinson’s disease and Mucinous ovarian cancer; Supplementary Figure S7: (A) Leave-one-out sensitivity analysis for Parkinson’s disease on the Low malignant potential ovarian cancer. (B) Scatter plot of the association between Parkinson’s disease and Low malignant potential ovarian cancer; Supplementary Figure S8: (A) Leave-one-out sensitivity analysis for Parkinson’s disease on the Low grade serous ovarian cancer. (B) Scatter plot of the association between Parkinson’s disease and Low grade serous ovarian cancer; Supplementary Figure S9: (A) Leave-one-out sensitivity analysis for Parkinson’s disease on the High grade serous ovarian cancer. (B) Scatter plot of the association between Parkinson’s disease and High grade serous ovarian cancer; Supplementary Figure S10: (A) Leave-one-out sensitivity analysis for Parkinson’s disease on the Endometrioid ovarian cancer. (B) Scatter plot of the association between Parkinson’s disease and Endometrioid ovarian cancer; Supplementary Figure S11: (A) Leave-one-out sensitivity analysis for Parkinson’s disease on the Clear cell ovarian cancer. (B) Scatter plot of the association between Parkinson’s disease and Clear cell ovarian cancer; Supplementary Tables S1–S12.
