# Supplementary material for: Ovarian Cancer and Parkinson’s Disease: A Bidirectional Mendelian Randomization Study
Source: J Clin Med. 2023 Apr 19;12(8):2961. doi: 10.3390/jcm12082961 (PMC10146810; doi:10.3390/jcm12082961)
Supplement: Supplementary file 1 [file jcm-12-02961-s001.zip › Supplementary Figure 4.pdf]

Supplementary Material

Supplementary Figures

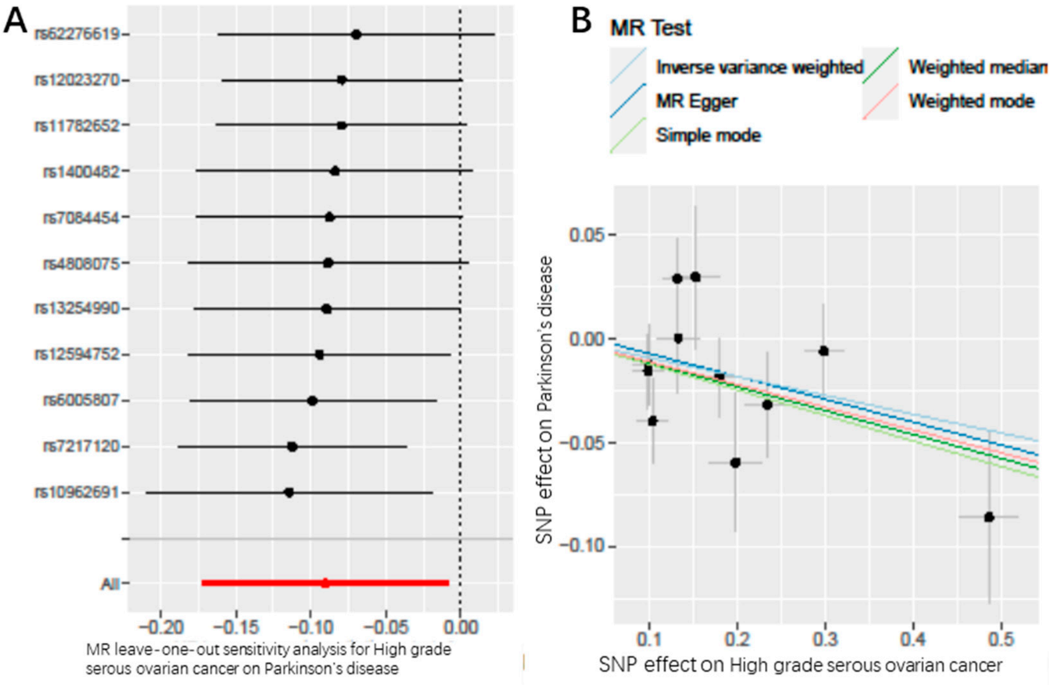

Supplementary Figure 4. (A) Leave-one-out sensitivity analysis for High grade serous ovarian cancer on the Parkinson's disease. **(B)** Scatter plot of the association between High grade serous ovarian cancer and Parkinson's disease.
